# Systemic Immune-Inflammation Markers Associate With Cervical Human Papillomavirus Infection in Asymptomatic Women

**DOI:** 10.1093/ofid/ofag194

**Published:** 2026-04-07

**Authors:** Myung-Gun Cho, Yoosoo Chang, Seungho Ryu

**Affiliations:** Department of Clinical Research Design and Evaluation, Samsung Advanced Institute for Health Sciences and Technology, Sungkyunkwan University, Seoul, South Korea; Department of Clinical Research Design and Evaluation, Samsung Advanced Institute for Health Sciences and Technology, Sungkyunkwan University, Seoul, South Korea; Center for Cohort Studies, Total Healthcare Center, Kangbuk Samsung Hospital, Sungkyunkwan University School of Medicine, Seoul, South Korea; Department of Occupational and Environmental Medicine, Kangbuk Samsung Hospital, Sungkyunkwan University School of Medicine, Seoul, South Korea; Department of Clinical Research Design and Evaluation, Samsung Advanced Institute for Health Sciences and Technology, Sungkyunkwan University, Seoul, South Korea; Center for Cohort Studies, Total Healthcare Center, Kangbuk Samsung Hospital, Sungkyunkwan University School of Medicine, Seoul, South Korea; Department of Occupational and Environmental Medicine, Kangbuk Samsung Hospital, Sungkyunkwan University School of Medicine, Seoul, South Korea

**Keywords:** women's health screening, human papillomavirus, leukocytes, cervical cytology, monocytes, systemic immune-inflammation markers

## Abstract

**Background:**

Human papillomavirus (HPV), a major cause of cervical cancer, typically remains localized to the cervix. However, there is limited understanding of whether this localized infection is associated with systemic immune responses in asymptomatic women, beyond the immediate cervical microenvironment. This study aimed to assess whether systemic immune-inflammation markers are linked to HPV infection and HPV-related cytological abnormalities in asymptomatic women undergoing health screening.

**Methods:**

This cross-sectional study included women from the Kangbuk Samsung Health Study who underwent HPV DNA testing and cervical cytology between 2016 and 2022. Participants were categorized as H0C0 (HPV-negative and normal cytology), H1C0 (HPV-positive and normal cytology), and H1C1 (HPV-positive and abnormal cytology, defined as low-grade abnormalities, including atypical squamous cells of undetermined significance and low-grade squamous intraepithelial lesions). Systemic immune-inflammation markers, including white blood cell–derived indices, high-sensitivity C-reactive protein, systemic inflammation response index (SIRI), lymphocyte-to-monocyte ratio (LMR), and neutrophil-to-monocyte ratio (NMR), were analyzed. Multinomial logistic regression was used to estimate the odds ratios (ORs) by biomarker quintiles and per 1-standard deviation (SD) increase.

**Results:**

Among 145 889 women (mean age 40.4 years), 6535 were classified as H1C0 and 2820 were classified as H1C1. Individuals with higher monocyte counts (Q5 vs Q1) had a significantly higher risk of H1C0 (OR = 1.14, 95% confidence interval [CI]: 1.06–1.24) and H1C1 (OR = 1.22, 95% CI: 1.08–1.37). Similarly, SIRI was positively associated with HPV infection status, with increasing odds for both H1C0 and H1C1 compared with H0C0, whereas LMR and NMR were inversely associated with H1C1. Per 1-SD increase, LMR was inversely associated with both H1C0 (OR = 0.71, 95% CI: 0.59–0.87) and H1C1 (OR = 0.48, 95% CI: 0.35–0.65), while monocyte count showed a positive association.

**Conclusions:**

Human papillomavirus infection was associated with systemic immune–inflammatory changes, most notably elevated monocyte count and related indices, even in individuals without cytological abnormalities. These findings highlight the potential relevance of monocyte-related markers for capturing the systemic immune effects of HPV infection beyond localized cervical changes.

Human papillomavirus (HPV) is a sexually transmitted virus strongly associated with various cancers, including cervical, head, neck, and anal cancers [1]. Cervical cancer is the fourth leading cause of cancer-related mortality in women worldwide, with over 95% of the cases being attributed to HPV infection [2]. More than 200 genotypes of HPV have been identified, some of which, such as HPV16 and HPV18, are classified as high risk owing to their oncogenic potential [3]. Persistent cervical infection with these high-risk genotypes can lead to cervical cancer, with high-grade lesions representing a later outcome of sustained viral persistence. However, the majority of HPV infections are transient and are spontaneously cleared by the host immune system [4]. Therefore, systemic immune alterations observed in asymptomatic HPV-positive women are more likely to reflect differences in host immune susceptibility and function rather than indicators of active disease progression.

Although HPV infection is typically localized to the cervix, inflammatory mediators produced in the infected microenvironment may enter the systemic circulation [5, 6]. Consequently, this localized immune response can alter the profile of circulating immune cells even in asymptomatic women. Emerging evidence indicates that HPV infection is associated with systemic inflammation, which has been implicated in diseases including atherosclerotic cardiovascular disease [7, 8]. However, it remains unclear whether systemic inflammation is associated with the direct viral presence or indirectly mediated by HPV-induced local inflammation, highlighting a critical gap in our understanding of the broader impact of HPV. Previous studies, primarily focusing on cytokine levels and other biomarkers in high-grade cervical intraepithelial neoplasia or cervical cancer, reported a positive association between HPV infection and systemic inflammation [5, 9–11]. Circulating markers, such as white blood cell (WBC) count, WBC-derived indices, and high-sensitivity C-reactive protein (hs-CRP), are well-established indicators of systemic inflammation [12, 13]. These markers can be routinely measured via standard blood tests, making them cost-effective and suitable for large-scale epidemiologic studies [13, 14]. However, cytokine testing is expensive, lacks standardization, and is not feasible for large-scale screening. Moreover, the association of HPV infection with systemic inflammation in women testing negative for intraepithelial lesion or malignancy or harboring low-grade lesions remains poorly understood. Human papillomavirus infection with normal cytology or low-grade lesions does not require immediate medical interventions [15, 16]; however, a subset of these women may develop persistent infection, which can subsequently progress to higher-grade intraepithelial lesions. Therefore, understanding the association between early-stage HPV infection and systemic immune-inflammatory status may provide important insights into host immune responses influencing viral persistence and disease progression.

Herein, the association between HPV infection and HPV-related cervical abnormalities and the systemic immune-inflammatory status was evaluated in asymptomatic women undergoing health screening, aiming to provide insights into potential pathways linking HPV infection with systemic inflammation.

## METHODS

### Study Population

This study was performed based on data of the Kangbuk Samsung Health Study, a cohort study of South Korean adults who participated in annual or biennial health examinations at the Kangbuk Samsung Hospital Total Healthcare Centers in Seoul and Suwon, South Korea [17]. This study was approved by the Institutional Review Board of the Kangbuk Samsung Hospital (IRB no. KBSMC 2023-03-039).

**Figure 1.**
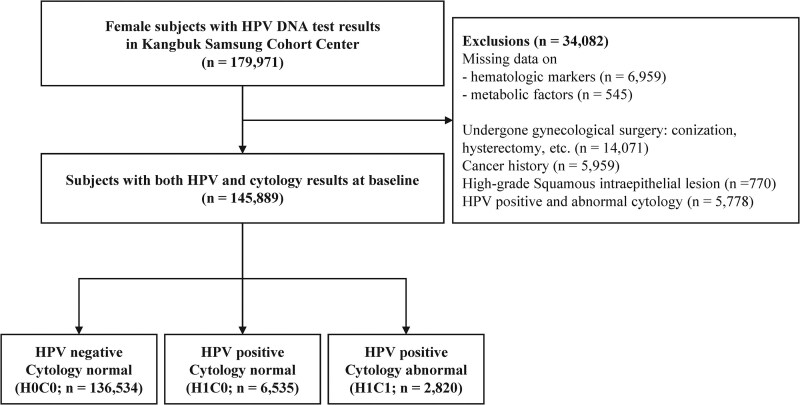
Study cohort selection protocol and distribution. Abbreviations: HPV, human papillomavirus; H0C0, HPV-negative with normal cytology; H1C0, HPV-positive with normal cytology; H1C1, HPV-positive with abnormal cytology.

The eligible study population comprised 179 971 women who underwent HPV DNA and cytology testing from 2016 to 2022. Participants were employees attending routine, legally mandated health screenings at a Total Healthcare Center under South Korea's Industrial Safety and Health Law and were not presenting with gynecological symptoms or complaints. High-risk HPV testing was performed as part of employer-contracted screening packages, independent of symptom status. We excluded participants with missing data on hematological markers and metabolic factors, a history of gynecological surgery (such as conization or hysterectomy), a history of cancer, or the presence of a high-grade cervical intraepithelial lesion. Additionally, individuals with HPV-negative results but abnormal cytology were excluded for 2 reasons: (1) cytological abnormalities in HPV-negative women are diagnostically heterogeneous, potentially arising from reactive changes, hormonal effects, or nonviral infections, precluding meaningful comparison with HPV-positive groups without additional microbiological characterization and (2) cervical inflammation may compromise cell viability during sampling, leading to false-negative HPV results [18], such that the H0C1 group may not reliably represent truly HPV-negative status. Consequently, a total of 34 082 participants were excluded based on the exclusion criteria, and 145 889 participants were included in this study (Figure 1).

### Data Collection

Data from self-reported sociodemographic factors, health behaviors, medical history, HPV DNA tests, cervical cytology tests, and laboratory measurements were collected. Smoking status was categorized as none, former, or current. Alcohol consumption was classified as none, moderate (<20 g per day), or high (≥20 g per day). Educational level was divided into low (less than an undergraduate degree) and high (an undergraduate degree or higher). Menopause was defined as the absence of menstruation for at least 1 year. Human papillomavirus vaccination status was self-reported. Trained nurses measured anthropometric parameters (blood pressure and body mass index). Blood samples were collected after 10-hour fasting for the measurement of glucose, lipid profile, insulin, and hs-CRP. Insulin resistance was determined using the homeostatic model assessment for insulin resistance (HOMA-IR) equation [19]. Metabolic unhealthy status was defined as ≥1 of the following: elevated glucose levels (≥100 mg/dL), high blood pressure (systolic ≥130 mm Hg or diastolic ≥85 mm Hg), triglycerides ≥150 mg/dL, high-density lipoprotein cholesterol <50 mg/dL, or HOMA-IR ≥2.5 [19–21], as well as the history of hypertension, diabetes, or hyperlipidemia and use of related medications.

Human papillomavirus DNA and cytology tests were conducted as part of a cervical cancer screening program. Cervical specimens were collected by medical specialists and analyzed at the core laboratory facility of the Kangbuk Samsung Hospital. The HPV DNA test results were reported as either negative or positive for HPV16 and HPV18 and for pooled results of 12 other high-risk HPV types (HPV31, HPV33, HPV35, HPV39, HPV45, HPV51, HPV52, HPV56, HPV58, HPV59, HPV66, and HPV68) using the Cobas HPV assay (Roche Diagnostics, Basel, Switzerland). The reference status (H0C0) comprised HPV negative and normal cytology, whereas H1C0 was HPV positive with normal cytology, and H1C1 was HPV positive with low-grade abnormal cytology.

The systemic immune-inflammation status was assessed using the following WBC-derived markers: systemic inflammation response index (SIRI = neutrophil × monocyte/lymphocyte), systemic immune-inflammation index (SIII = neutrophil × platelet/lymphocyte), neutrophil-to-lymphocyte ratio (NLR), lymphocyte-to-monocyte ratio (LMR), neutrophil-to-monocyte ratio (NMR), platelet-to-lymphocyte ratio (PLR), WBC subtypes (neutrophil, lymphocyte, monocyte, eosinophil, and basophil), platelets, and hs-CRP. The WBC and differential counts were measured using an XE-2100 hematology analyzer (Sysmex Corporation, Kobe, Japan) [22]. The absolute WBC subtype count was calculated by multiplying the total WBC count by the percentage of each subtype.

### Statistical Analysis

The collected data are presented as mean ± standard deviation (SD) or median (interquartile range) for continuous variables and as proportions for categorical variables. Systemic immune-inflammation markers were divided into quintiles based on their distribution in the overall study population (quintile cutoff values are summarized in Supplementary Table 1).

The baseline levels of systemic immune-inflammation markers were compared among the H0C0, H1C0, and H1C1 groups, with the H0C0 group serving as reference. To assess the relationship between HPV-cytology status and systemic immune-inflammation markers, multinomial logistic regression models were used to calculate odds ratios (ORs) with 95% confidence intervals (CIs) for comparing Q2–Q5 with Q1 and provide an overall comparison across quintiles. In addition, to allow comparisons across biomarkers with different units and distributions, each marker was standardized, and ORs were estimated per 1-SD increase to evaluate linear associations with HPV-cytology status. Three models were fitted with progressive adjustments for potential confounding factors. Model 1 was a crude model. Model 2 was adjusted for age, smoking status, alcohol intake, education level, study center, menopausal status, hs-CRP level, and metabolic health status. Model 3 was additionally adjusted for the HPV vaccination status. In the sensitivity analysis, the relative instead of the absolute counts of WBC subtypes were used, which were then categorized into quintiles. To minimize the risk of type I errors from multiple comparisons, we applied the Bonferroni correction. All statistical analyses were performed using R (version 4.5.1).

## RESULTS

### Baseline Characteristics of the Study Population

The 145 889 participants (mean age: 40.4 years) were categorized based on their HPV DNA test and cervical cytology results (Table 1). During their first visit, 93.6%, 4.5%, and 1.9% of the participants were classified as normal (H0C0), HPV positive with normal cytology (H1C0), and HPV positive with low-grade lesions (H1C1), respectively.

**Table 1.** Baseline Characteristics of the Study Cohort According to Human Papillomavirus (HPV) DNA and Cytology Testing Results

Variable	Total (n = 145 889)	H0C0 (n = 136 534)	H1C0 (n = 6535)	H1C1 (n = 2820)	*P*-Value^a^
Age (mean ± SD)	40.4 ± 10.3	40.6 ± 10.3	38.1 ± 10.7	36.4 ± 9.0	<.001
Smoke (%)					<.001
None	90.3	90.5	87.8	87.1	
Former	7.0	7.0	7.6	8.3	
Smoker	1.9	1.8	3.5	3.9	
Unknown	0.8	0.7	1.1	0.7	
Alcohol (%)^b^					
None	24.8	25.3	18.4	15.7	<.001
Moderate	64.3	64.1	66.4	68.3	
High (≥20 g/d)	5.1	4.7	10.7	12.2	
Unknown	5.8	5.9	4.5	3.8	
Education level					<.001
Low	37.6	37.2	43.7	44.5	
High (above undergraduate)	59.6	60.0	53.2	53.5	
Unknown	2.8	2.8	3.1	2.0	
Metabolic health^b^ (yes, %)	88.4	88.2	90.6	92.0	<.001
Postmenopausal (yes, %)	15.0	15.3	13.6	8.0	<.001
HPV vaccination (yes, %)	36.1	35.7	40.8	41.8	<.001
Center (%)					.181
Seoul	45.1	45.1	45.0	43.4	
Suwon	54.9	54.9	55.0	56.6	
HPV type (n)					<.001
HPV16	1087	…	739	348	
HPV18	490	…	349	141	
HPV others	8203	…	5671	2532	
SIRI (N × M/L)^c^	525.3 (364.6–760.3)	533.4 (363.1–769.3)	548.0 (382.0–804.0)	526.0 (364.8–761.5)	<.001
SIII (N × P/L)^c^	412.9 (305.8–560.8)	408.4 (301.5–560.7)	421.1 (312.1–572.0)	412.8 (305.7–561.0)	.032
NLR (N/L)^c^	1.6 (1.2–2.0)	1.6 (1.2–2.0)	1.6 (1.2–2.1)	1.6 (1.2–2.0)	.012
LMR (L/M)^c^	5.8 (4.6–7.2)	5.7 (4.5–7.2)	5.6 (4.4–7.0)	5.8 (4.6–7.2)	<.001
NMR (N/M)^c^	9.2 (7.3–11.5)	9.0 (7.1–11.3)	9.1 (7.2–11.4)	9.1 (7.3–11.5)	<.001
PLR (P/L)^c^	135.8 (112.3–165.0)	136.0 (111.8–165.6)	136.4 (113.3–165.7)	135.8 (112.3–165.1)	.843
WBC (cells/μL)^c^	5540.0 (4670.0–6580.0)	5530.0 (4640.0–6590.0)	5625.0 (4740.0–6590.0)	5540.0 (4670.0–6580.0)	.105
Neutrophil (cells/μL)^c^	3050.9 (2397.8–3870.9)	3016.8 (2375.5–3905.1)	3110.8 (2449.9–3947.8)	3050.3 (2397.8–3874.3)	.024
Lymphocyte (cells/μL)^c^	1924.3 (1619.1–2279.4)	1928.9 (1618.9–2279.6)	1909.4 (1618.3–2270.9)	1923.2 (1619.1–2279.4)	.697
Monocyte (cells/μL)^c^	333.0 (270.3–411.8)	340.2 (271.7–421.0)	341.9 (278.4–427.4)	337.1 (270.5–412.2)	<.001
Eosinophil (cells/μL)^c^	98.7 (59.0–161.2)	93.1 (58.2–160.9)	92.8 (58.2–160.5)	98.6 (58.9–161.2)	.114
Basophil (cells/μL)^c^	21.6 (12.9–32.6)	21.4 (12.6–32.2)	21.1 (12.3–31.9)	21.6 (12.9–32.5)	.002
Platelet (×10^3^ cells/μL)^c^	262.0 (227.0–301.0)	262.0 (227.0–301.0)	262.0 (228.0–301.0)	262.0 (229.0–300.0)	.87
hs-CRP (mg/L)^c^	0.4 (0.2–0.7)	0.4 (0.2–0.7)	0.3 (0.2–0.7)	0.3 (0.2–0.6)	<.001

Abbreviations: SIRI, systemic inflammation response index; SIII, systemic immune-inflammation index; NLR, neutrophil-to-lymphocyte ratio; LMR, lymphocyte-to-monocyte ratio; NMR, neutrophil-to-monocyte ratio; PLR, platelet-to-lymphocyte ratio; WBC, white blood cell; hs-CRP, high-sensitivity C-reactive protein; H0C0, HPV-negative and normal cytology; H1C0, HPV-positive and normal cytology; H1C1, HPV-positive and abnormal cytology.

^a^The *P*–values were calculated using the Kruskal–Wallis test for continuous variables and the χ2 test for categorical variables.

^b^Metabolic health status, the following criteria must be met: glucose <100, blood pressure <135/85, triglycerides <150, high–density lipoprotein ≥50, HOMA-IR <2.5; no history of hypertension, diabetes, or hyperlipidemia, and no use of related medications.

^c^Values are represented as median (Q1–Q3).

The demographic characteristics associated with the sequential progression from H0C0 to H1C0 to H1C1 revealed a tendency toward younger age, higher smoking and alcohol consumption, and lower educational status. Additionally, the H1C1 group showed a lower rate of menopause, better metabolic health, and higher rate of HPV vaccination. Genotyping data revealed that HPV16 was present in 11.6% (1087/9355) of all HPV-positive individuals, including 8.4% cases (789/9355) as a single infection. The HPV18 was found in 5.4% (348/9355) of cases, with 4.3% cases (276/9355) as a single infection. Other high-risk HPV (HR-HPV) types were identified in 86.8% of the cases. Statistically significant differences were observed in certain WBC-derived indices and WBC subtypes, including the SIRI, SIII, NLR, LMR, NMR, neutrophils, monocytes, and basophils. In addition, hs-CRP levels were lower in the H1C0 and H1C1 groups.

### Association Between Systemic Immune-Inflammation Marker Quintiles and HPV-Cytology Status

Multinomial regression analyses were performed to assess the relationship between systemic immune-inflammation markers and the HPV-cytology status of the patients (Table 2 and Supplementary Table 2). Notably, SIRI was found to be significantly positively associated with HPV progression. Compared with the H0C0 group, the OR for the H1C1 group was significantly higher across all models, whereas no significant differences were observed in the H1C0 group. Moreover, both LMR and NMR showed significantly decreasing trends with the progression of HPV infection. Compared with the H0C0 group, the OR for LMR was lower in both the H1C0 and H1C1 groups, with a more pronounced decrease in H1C1 (OR & 0.88 [0.82, 0.95] for H1C0 and 0.73 [0.65, 0.82] for H1C1 in model 3). Similarly, NMR showed a significant decrease in both H1C0 and H1C1 groups (OR & 0.83 [0.77, 0.9] and 0.87 [0.77, 0.98], respectively, in model 3). No significant differences in the other WBC-derived indices were observed, including SIII, NLR, and PLR. Nonetheless, monocytes were associated to gradually higher ORs during HPV progression. Compared with the H0C0 group, the OR was higher in both the H1C0 (OR & 1.14 [1.06, 1.24] in model 3) and H1C1 (OR & 1.22 [1.08, 1.37] in model 3) groups, with a more pronounced increase in H1C1. Additionally, slightly lower ORs were observed for lymphocytes, eosinophils, and basophils. No significant differences in other WBC subtypes, including total WBC count, neutrophils, and platelet counts, were observed. The hs-CRP levels showed a negative association with HPV infection progression from H0C0 to H1C0 and H1C1 (OR & 0.85 [0.77, 0.94] for H1C0 and 0.71 [0.61, 0.82] for H1C1 in model 3).

**Table 2.** Odds Ratios (ORs) for Systemic Immune-Inflammation Markers According to HPV-Cytology Status at Baseline

Systemic Immune-Inflammation Markers	HPV and Cytology Group	ORs (95% Confidence Interval) (Q5 vs Q1, Reference: Q1)		
		Model 1	Model 2	Model 3
SIRI	H0C0	Ref.	Ref.	Ref.
	H1C0	1.03 (.95–1.11)	1.03 (.95–1.11)	1.03 (.95–1.11)
	H1C1	1.3 (1.16–1.47)^a^	1.24 (1.1–1.4) ^a^	1.24 (1.1–1.4) ^a^
SIII	H0C0	Ref.	Ref.	Ref.
	H1C0	0.95 (.88–1.03)	0.94 (.87–1.02)	0.94 (.87–1.02)
	H1C1	1.1 (.98–1.24)	1.03 (.91–1.16)	1.02 (.91–1.16)
NLR	H0C0	Ref.	Ref.	Ref.
	H1C0	0.95 (.88–1.02)	0.96 (.89–1.04)	0.96 (.89–1.04)
	H1C1	1.14 (1.02–1.28)	1.11 (.99–1.25)	1.11 (.99–1.25)
LMR	H0C0	Ref.	Ref.	Ref.
	H1C0	0.92 (.85–.99)	0.88 (.82–.95)^a^	0.88 (.82–.95)^a^
	H1C1	0.73 (.65–.82)^a^	0.73 (.64–.82)^a^	0.73 (.65–.82)^a^
NMR	H0C0	Ref.	Ref.	Ref.
	H1C0	0.85 (.79–.92) ^a^	0.83 (.77–.9) ^a^	0.83 (.77–.9) ^a^
	H1C1	0.9 (.8–1.01)	0.87 (.77–.98)	0.87 (.77–.98)
PLR	H0C0	Ref.	Ref.	Ref.
	H1C0	0.99 (.92–1.07)	1.02 (.94–1.1)	1.02 (.94–1.1)
	H1C1	1.02 (.91–1.15)	1.01 (.9–1.14)	1.01 (.89–1.14)
WBC	H0C0	Ref.	Ref.	Ref.
	H1C0	1 (.92–1.08)	0.95 (.87–1.02)	0.95 (.87–1.02)
	H1C1	1.11 (.98–1.25)	1.01 (.89–1.14)	1.01 (.89–1.14)
Neutrophil	H0C0	Ref.	Ref.	Ref.
	H1C0	1 (.92–1.08)	0.97 (.89–1.05)	0.97 (.89–1.05)
	H1C1	1.14 (1.01–1.28)	1.05 (.93–1.19)	1.05 (.93–1.19)
Lymphocyte	H0C0	Ref.	Ref.	Ref.
	H1C0	0.96 (.88–1.03)	0.89 (.82–.96)	0.89 (.82–.96)
	H1C1	0.96 (.85–1.08)	0.89 (.79–1)	0.89 (.79–1)
Monocyte	H0C0	Ref.	Ref.	Ref.
	H1C0	1.16 (1.07–1.25)^a^	1.14 (1.06–1.24)^a^	1.14 (1.06–1.24)^a^
	H1C1	1.29 (1.14–1.45)^a^	1.22 (1.08–1.37)^a^	1.22 (1.08–1.37)^a^
Eosinophil	H0C0	Ref.	Ref.	Ref.
	H1C0	0.96 (.89–1.04)	0.92 (.85–1)	0.92 (.85–1)
	H1C1	0.94 (.83–1.05)	0.87 (.77–.98)	0.87 (.78–.98)
Basophil	H0C0	Ref.	Ref.	Ref.
	H1C0	0.9 (.83–.97)	0.91 (.84–.98)	0.91 (.84–.98)
	H1C1	0.88 (.78–.99)	0.89 (.79–1)	0.89 (.79–1)
Platelet	H0C0	Ref.	Ref.	Ref.
	H1C0	0.99 (.91–1.07)	0.95 (.87–1.02)	0.95 (.87–1.02)
	H1C1	0.99 (.87–1.11)	0.89 (.79–1.01)	0.89 (.79–1.01)
hs-CRP	H0C0	Ref.	Ref.	Ref.
	H1C0	0.8 (.73–.88)^a^	0.85 (.77–.94)^a^	0.85 (.77–.94)^a^
	H1C1	0.63 (.55–.73)^a^	0.71 (.61–.82)^a^	0.71 (.61–.82)^a^

Abbreviations: SIRI, systemic inflammation response index; SIII, systemic immune-inflammation index; NLR, neutrophil-to-lymphocyte ratio; LMR, lymphocyte-to-monocyte ratio; NMR, neutrophil-to-monocyte ratio; PLR, platelet-to-lymphocyte ratio; WBC, white blood cells; hs-CRP, high-sensitivity C-reactive protein; H0C0, HPV-negative and normal cytology; H1C0, HPV-positive and normal cytology; H1C1, HPV-positive and abnormal cytology.

^a^Statistical significance after applying the Bonferroni correction.

In the sensitivity analysis, the relative values of monocytes, eosinophils, and basophils showed trends similar to those of the absolute counts. For the relative values of monocytes, the ORs were 1.21 [1.12, 1.31] for H1C0 and 1.34 [1.19, 1.51] for H1C1 in model 3.

### Association Between a per 1-SD Increase in Systemic Immune-Inflammation Markers and HPV-cytology Status

**Figure 2.**
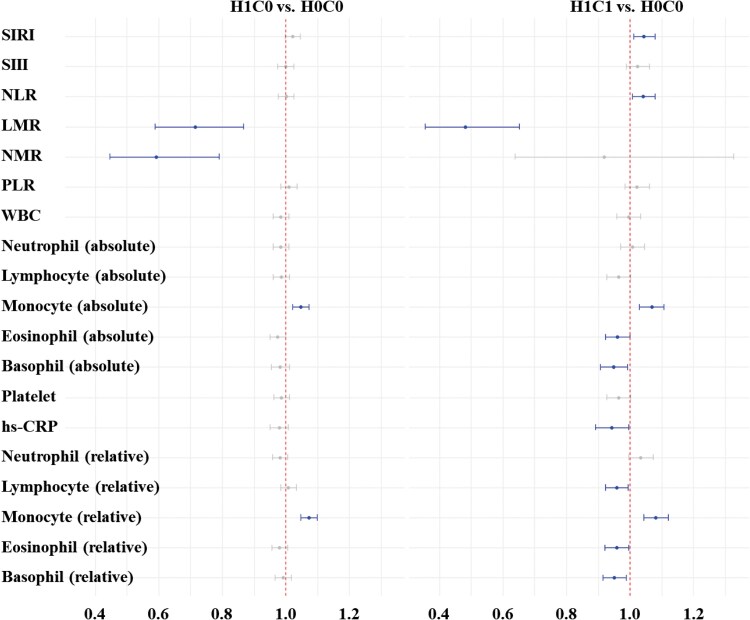
Forest plot of adjusted odds ratios and 95% confidence intervals for H1C0 and H1C1 versus H0C0 per 1-standard deviation increase in systemic immune-inflammation biomarkers. All abbreviations used are described in Table 2.

To explore linear associations, systemic immune-inflammation markers were standardized and ORs were estimated per 1-SD increase using multinomial logistic regression (Figure 2). Lymphocyte-to-monocyte ratio was inversely associated with HPV-cytology progression (OR = 0.71 [95% CI: 0.59–0.87] for H1C0 and OR = 0.48 [95% CI: 0.35–0.65] for H1C1). Neutrophil-to-monocyte ratio showed a significant inverse association only in the H1C0 group (OR = 0.59 [95% CI: 0.45–0.79]), with no statistically significant relationship observed for H1C1. Both monocyte count and monocyte ratio were positively associated with HPV-cytology progression in H1C0 and H1C1. Specifically, per 1-SD increase in absolute monocyte count, the ORs were 1.05 (95% CI: 1.02–1.07) for H1C0 and 1.07 (95% CI: 1.03–1.11) for H1C1. Likewise, per 1-SD increase in relative monocyte ratio, the ORs were 1.07 (95% CI: 1.05–1.10) for H1C0 and 1.08 (95% CI: 1.04–1.12) for H1C1.

## DISCUSSION

In this study, we used 2 complementary approaches to assess the relationship between systemic immune-inflammation markers and HPV-cytology status. First, in the cross-sectional analysis of prevalent cases, each biomarker was categorized into quintiles, and multinomial logistic regression was applied to estimate the ORs for H1C0 and H1C1 versus H0C0 across these quintile groups. Second, to assess linear associations, biomarkers were standardized and ORs per 1-SD increase were estimated. By combining categorical (quintile-based) and continuous (per-SD) frameworks, we captured both threshold effects and linear trends in how immune-inflammation markers relate to HPV infection and cervical cytology changes.

Statistically significant associations were observed between several systemic immune-inflammation markers and HPV-cytology status. Specifically, SIRI was positively associated with H1C1, whereas LMR and NMR were inversely associated with H1C0 and H1C1. Lymphocyte-to-monocyte ratio and NMR indices use monocytes as the denominator, whereas SIRI is determined using monocytes as numerator. In the absence of significant changes in major WBC subtypes, such as neutrophils and lymphocytes, it is likely that the influence of monocytes primarily drives the observed changes in WBC-derived indices. In sensitivity analysis using relative WBC values, monocytes remained significantly associated with HPV-cytology status, with higher ORs for both H1C0 and H1C1. Even in the per-1-SD increase analysis, monocyte-related markers were significantly associated with the HPV-cytology status.

The observed increase in circulating monocytes may reflect disruption of normal monocyte differentiation by HPV oncoproteins. E6 has been shown to inhibit the differentiation of monocytes into dendritic or Langerhans cells, potentially leading to an accumulation of undifferentiated monocytes in the peripheral blood [23–25].

Furthermore, E6 and E7 may impair monocyte trafficking by suppressing the expression of monocyte chemoattractant protein-1 (MCP-1), a key chemokine for recruiting monocytes to infected tissues [26], preventing monocyte migration to the cervix and resulting in their compensatory retention and increase in the circulation, consistent with previously reported reductions in MCP-1 in HPV-positive individuals [9]. Although specific cytokines were not directly measured in this study, these mechanisms provide a plausible biological explanation for our findings and warrant validation through future studies incorporating direct cytokine profiling.

For hs-CRP, a statistically significant decreasing trend is related to HPV infection and progression from baseline. No previous studies have directly examined the relationship between HPV and hs-CRP levels. The observed decrease in the hs-CRP levels with HPV infection and progression can be interpreted as follows: First, HPV is a localized infection that infects the basal cells of the epithelial tissues of the cervix, anus, and oral and oropharyngeal epithelia [27]. In acute systemic infections, such as influenza or bacterial infections, interleukin-6 is transported through the bloodstream to the liver, increasing hs-CRP levels. However, during HPV infection, interleukin-6 may only increase locally in the cervix without triggering a systemic hs-CRP response. Another possible explanation is that HPV employs immune evasion strategies to suppress systemic inflammatory responses. Human papillomavirus infection increases anti-inflammatory cytokines, such as interleukin-10 and transforming growth factor-β [28, 29], which may reduce hs-CRP levels. However, further studies are required to confirm this hypothesis.

In model 3 of the multinomial logistic regression, the HPV vaccination status was also considered in addition to the variables used in model 2, but no significant differences in the ORs were observed. This cross-sectional study lacked information on the timing of HPV vaccination and whether it occurred before or after infection. Following HPV vaccination, the levels of immunoglobulin G (IgG), IgM, and IgA, as well as HPV-specific memory B and T cells significantly increase, suggesting an increase in lymphocyte subpopulations related to the adaptive immune response [30, 31].

Given the large sample size of this study, the observed statistical significance should be interpreted with caution regarding clinical utility, as the effect sizes (ORs) were modest. Accordingly, monocyte counts or SIRI are not currently suitable as a standalone diagnostic or screening tool for HPV infection. Rather, the relevance of these findings is primarily pathophysiological—the consistent association between HPV status and elevated systemic inflammatory markers provides epidemiological evidence that HPV infection is associated with systemic immune alterations that extend beyond the localized cervical microenvironment, challenging the conventional view of HPV as a strictly localized mucosal infection.

The study had the following limitations. First, cross-sectional studies assess exposure and outcomes simultaneously, making it difficult to establish a cause-and-effect relationship. Therefore, further studies are needed to further validate the relationship between HPV-cytology status and systemic immune-inflammation markers. However, as HPV is an external pathogen transmitted through sexual contact, reverse causality is considered minimal. Second, information regarding sexual history and co-infections was not available. We acknowledge that systemic inflammation could be influenced by unmeasured coinfections associated with high-risk behaviors. However, South Korea has a remarkably low prevalence of sexually transmitted infections (STIs) even for the most common ones—genital herpes, the most prevalent STI, was only ∼0.25% and HIV at ∼0.02% in the general population [32]—making it unlikely that undiagnosed co-infections substantially confounded our results. Additionally, although categorical adjustment for smoking and alcohol was applied in our multivariate models, residual confounding from smoking or alcohol dosage remains a theoretical concern. However, given the very low prevalence of current smoking (1.9%) and high-risk drinking (5.1%) in our cohort, the practical impact of this limitation is likely minimal. Furthermore, subgroup analyses restricted to noncurrent smokers and nonheavy drinkers yielded consistent findings (Supplementary Table 3), suggesting that behavioral factors are unlikely to fully account for the observed associations between HPV infection and monocyte-related markers, although residual confounding cannot be entirely excluded. Nevertheless, our findings should be interpreted as associations within a low-risk screening population, and future prospective studies incorporating sexual history and STI screening are warranted. Finally, information on high-risk HPV genotypes other than HPV16 and HPV18 was not available, indicating that genotype-specific risk attribution was not reflected in the analysis.

In conclusion, our results suggest that HPV infection is associated with systemic immune-inflammation alterations, namely elevated monocyte counts and derived indices, even in individuals without cytological abnormalities. These findings underscore the potential utility of monocyte-related biomarkers as early indicators of HPV-associated immune alterations and highlight that HPV infection is associated with systemic immune responses that extend beyond localized cervical pathology. Our results align with the emerging evidence that HPV is linked to systemic immunological effects in addition to its established local impacts. Future investigations should determine whether these inflammatory markers can predict the risk of disease progression and assess their normalization following successful viral clearance.

## Supplementary Material

ofag194_Supplementary_Data

## Supplementary Data

Supplementary materials are available at *Open Forum Infectious Diseases* online. Consisting of data provided by the authors to benefit the reader, the posted materials are not copyedited and are the sole responsibility of the authors, so questions or comments should be addressed to the corresponding author.

## References

[1] WHO . Human papillomavirus and cancer. **2024**; Available at: https://www.who.int/news-room/fact-sheets/detail/human-papilloma-virus-and-cancer. Accessed 04 November 2025.

[2] Tanzi E, Canuti M. HPV infection and cervical cancer. In: Raviglione MCB et al, ed. Global health essentials. Cham: Springer International Publishing, 2023:109–16.

[3] Viveros-Carreño D, Fernandes A, Pareja R. Updates on cervical cancer prevention. Int J Gynecol Cancer 2023; 33:394–402. 36878567 10.1136/ijgc-2022-003703

[4] Kiamba EW, Goodier MR, Clarke E. Immune responses to human papillomavirus infection and vaccination. Front Immunol 2025; 16:1591297. 40589751 10.3389/fimmu.2025.1591297 PMC12206648

[5] Vitkauskaite A, Urboniene D, Celiesiute J, et al Circulating inflammatory markers in cervical cancer patients and healthy controls. J Immunotoxicol 2020; 17:105–9. 32364810 10.1080/1547691X.2020.1755397

[6] Scott ME, Shvetsov YB, Thompson PJ, et al Cervical cytokines and clearance of incident human papillomavirus infection: Hawaii HPV cohort study. Int J Cancer 2013; 133:1187–96. 23436563 10.1002/ijc.28119 PMC3732505

[7] Chan NC, Lawson JS, Hirsh J. Human papilloma virus and atherosclerotic cardiovascular disease. Eur Heart J 2024; 45:1083–5. 38321363 10.1093/eurheartj/ehad829

[8] Dutta P, Saha D, Earle M, et al Unveiling HPV's hidden link: cardiovascular diseases and the viral intrigue. Indian Heart J 2024; 76:1–5. 38387552 10.1016/j.ihj.2024.02.001 PMC10943540

[9] Olukomogbon T, Akpobome B, Omole A, Adebamowo CA, Adebamowo SN. Association between cervical inflammatory mediators and prevalent cervical human papillomavirus infection. JCO Glob Oncol 2024; 10:e2300380. 38547441 10.1200/GO.23.00380 PMC10994421

[10] Hong JH, Kim MK, Lee IH, et al Association between Serum cytokine profiles and clearance or persistence of high-risk human papillomavirus infection: a prospective study. Int J Gynecol Cancer 2010; 20:1011. 20683410 10.1111/IGC.0b013e3181e513e5

[11] Bonin-Jacob CM, Almeida-Lugo LZ, Puga MAM, et al IL-6 and IL-10 in the serum and exfoliated cervical cells of patients infected with high-risk human papillomavirus. PLoS One 2021; 16:e0248639. 33750983 10.1371/journal.pone.0248639 PMC7984643

[12] Kounis NG, Soufras GD, Tsigkas G, Hahalis G. White blood cell counts, leukocyte ratios, and eosinophils as inflammatory markers in patients with coronary artery disease. Clin Appl Thromb Hemost 2014; 21:139–43. 24770327 10.1177/1076029614531449

[13] Pandya D, Bhetariya B, Koitiya N. High-sensitivity C-reactive protein as a biomarker of cardiovascular events. Natl J Physiol Pharm Pharmacol 2023; 14: 210–2

[14] Seo I-H, Lee Y-J. Usefulness of complete blood count (CBC) to assess cardiovascular and metabolic diseases in clinical settings: a comprehensive literature review. Biomedicines 2022; 10:2697. 36359216 10.3390/biomedicines10112697 PMC9687310

[15] Fontham ETH, Wolf AMD, Church TR, et al Cervical cancer screening for individuals at average risk: 2020 guideline update from the American Cancer Society. CA Cancer J Clin 2020; 70:321–46. 32729638 10.3322/caac.21628

[16] Pimple SA, Mishra GA. Global strategies for cervical cancer prevention and screening. Minerva Ginecol 2019; 71:313–20. 30808155 10.23736/S0026-4784.19.04397-1

[17] Cho Y, Chang Y, Ryu S, Wild SH, Byrne CD. Baseline and change in serum uric acid level over time and resolution of nonalcoholic fatty liver disease in young adults: the Kangbuk Samsung Health Study. Diabetes Obes Metab 2024; 26:1644–57. 38303100 10.1111/dom.15466

[18] Xing B, Guo J, Sheng Y, Wu G, Zhao Y. Human papillomavirus-negative cervical cancer: a comprehensive review. Front Oncol 2020; 10:606335. 33680928 10.3389/fonc.2020.606335 PMC7925842

[19] Chang Y, Kim B-K, Yun KE, et al Metabolically-healthy obesity and coronary artery calcification. J Am Coll Cardiol 2014; 63:2679–86. 24794119 10.1016/j.jacc.2014.03.042

[20] Chang Y, Ryu S, Choi Y, et al Metabolically healthy obesity and development of chronic kidney disease: a cohort study. Ann Intern Med 2016; 164:305–12. 26857595 10.7326/M15-1323

[21] Lee J, Kim HS, Kim K, Bae D-S, Kim B-G, Choi CH. Metabolic syndrome and persistent cervical human papillomavirus infection. Gynecol Oncol 2021; 161:559–64. 33676760 10.1016/j.ygyno.2021.02.009

[22] Ruzicka K, Veitl M, Thalhammer-Scherrer R, Schwarzinger I. The new hematology analyzer Sysmex XE-2100: performance evaluation of a novel white blood cell differential technology. Arch Pathol Lab Med 2001; 125:391–6. 11231489 10.5858/2001-125-0391-TNHASX

[23] Della Fera AN, Warburton A, Coursey TL, Khurana S, McBride AA. Persistent human papillomavirus infection. Viruses 2021; 13:321. 33672465 10.3390/v13020321 PMC7923415

[24] Kusakabe M, Taguchi A, Sone K, Mori M, Osuga Y. Carcinogenesis and management of human papillomavirus-associated cervical cancer. Int J Clin Oncol 2023; 28:965–74. 37294390 10.1007/s10147-023-02337-7 PMC10390372

[25] Iijima N, Goodwin EC, DiMaio D, Iwasaki A. High-risk human papillomavirus E6 inhibits monocyte differentiation to Langerhans cells. Virology 2013; 444:257–62. 23871219 10.1016/j.virol.2013.06.020 PMC3755085

[26] Kleine-Lowinski K, Rheinwald JG, Fichorova RN, et al Selective suppression of monocyte chemoattractant protein-1 expression by human papillomavirus E6 and E7 oncoproteins in human cervical epithelial and epidermal cells. Int J Cancer 2003; 107:407–15. 14506741 10.1002/ijc.11411

[27] Wolf J, Kist LF, Pereira SB, et al Human papillomavirus infection: epidemiology, biology, host interactions, cancer development, prevention, and therapeutics. Rev Med Virol 2024; 34:e2537. 38666757 10.1002/rmv.2537

[28] Fernandes JV, De Medeiros Fernandes TAA, De Azevedo JCV, et al Link between chronic inflammation and human papillomavirus-induced carcinogenesis (review). Oncol Lett 2015; 9:1015–26. 25663851 10.3892/ol.2015.2884 PMC4315066

[29] Mangino G, Chiantore MV, Iuliano M, Fiorucci G, Romeo G. Inflammatory microenvironment and human papillomavirus-induced carcinogenesis. Cytokine Growth Factor Rev 2016; 30:103–11. 27021827 10.1016/j.cytogfr.2016.03.007

[30] Yokomine M, Matsueda S, Kawano K, et al Enhancement of humoral and cell mediated immune response to HPV16 L1-derived peptides subsequent to vaccination with prophylactic bivalent HPV L1 virus-like particle vaccine in healthy females. Exp Ther Med 2017; 13:1500–5. 28413500 10.3892/etm.2017.4150 PMC5377531

[31] Pasmans H, Berkowska MA, Diks AM, et al Characterization of the early cellular immune response induced by HPV vaccines. Front Immunol 2022; 13:863164. 35924247 10.3389/fimmu.2022.863164 PMC9341268

[32] Song JY, Kim KS, Han CH, Bae S. Recent changes in sexually transmitted infection in Korea: a population-based analysis. J Clin Med 2025; 14:5145. 40725838 10.3390/jcm14145145 PMC12295904

