# Supplementary material for: Systemic Immune-Inflammation Markers Associate With Cervical Human Papillomavirus Infection in Asymptomatic Women
Source: Open Forum Infect Dis. 2026 Apr 7;13(4):ofag194. doi: 10.1093/ofid/ofag194 (PMC13075954; doi:10.1093/ofid/ofag194)
Supplement: ofag194_Supplementary_Data [file ofag194_supplementary_data.docx]

Supplementary Table 1. Quintile cut-off values for systemic immune-inflammation markers

| Marker | Q1/Q2 | Q2/Q3 | Q3/Q4 | Q4/Q5 |
| --- | --- | --- | --- | --- |
| SIRI | 333.5 | 458.9 | 603.5 | 836.0 |
| SIII | 283.4 | 369.0 | 462.9 | 606.6 |
| NLR | 1.2 | 1.4 | 1.7 | 2.2 |
| LMR | 4.4 | 5.3 | 6.3 | 7.6 |
| NMR | 6.9 | 8.4 | 9.9 | 12.1 |
| PLR | 107.0 | 126.4 | 145.9 | 173.5 |
| WBC | 4480 | 5200 | 5910 | 6870 |
| Neutrophil | 2257.8 | 2788.8 | 3339.0 | 4108.5 |
| Lymphocyte | 1549.5 | 1806.8 | 2050.7 | 2376.9 |
| Monocyte | 258.3 | 309.7 | 361.7 | 436.1 |
| Eosinophil | 50.3 | 80.2 | 118.8 | 182.4 |
| Basophil | 11.4 | 19.8 | 28.4 | 39.0 |
| Platelet | 219,000 | 248,000 | 276,000 | 312,000 |
| hs-CRP | 0.2 | 0.3 | 0.4 | 0.9 |

Supplementary Table 2. Odds Ratios (ORs) for systemic immune-inflammatory markers by HPV and cytology status at baseline

| Marker | Comparison Group | Model1 | | | Model2 | | | Model3 | | |
| --- | --- | --- | --- | --- | --- | --- | --- | --- | --- | --- |
|  |  | Quintile | OR (95% CI) | *p*-value | Quintile | OR (95% CI) | *p*-value | Quintile | OR (95% CI) | *p*-value |
| SIRI | H1C0 | Q1 | 1.00 (Reference) | - | Q1 | 1.00 (Reference) | - | Q1 | 1.00 (Reference) | - |
|  |  | Q2 | 0.95 (0.87-1.02) | 0.169 | Q2 | 0.96 (0.88-1.04) | 0.271 | Q2 | 0.96 (0.88-1.04) | 0.269 |
|  |  | Q3 | 0.96 (0.88-1.03) | 0.265 | Q3 | 0.97 (0.90-1.05) | 0.496 | Q3 | 0.97 (0.90-1.05) | 0.494 |
|  |  | Q4 | 1.00 (0.92-1.08) | 0.956 | Q4 | 1.01 (0.94-1.10) | 0.752 | Q4 | 1.01 (0.94-1.10) | 0.754 |
|  |  | Q5 | 1.03 (0.95-1.11) | 0.493 | Q5 | 1.03 (0.95-1.11) | 0.455 | Q5 | 1.03 (0.95-1.11) | 0.454 |
| SIRI | H1C1 | Q1 | 1.00 (Reference) | - | Q1 | 1.00 (Reference) | - | Q1 | 1.00 (Reference) | - |
|  |  | Q2 | 1.12 (0.99-1.27) | 0.070 | Q2 | 1.11 (0.98-1.26) | 0.086 | Q2 | 1.12 (0.99-1.26) | 0.085 |
|  |  | Q3 | 1.21 (1.07-1.36) | 0.003 | Q3 | 1.19 (1.05-1.35) | 0.005 | Q3 | 1.19 (1.05-1.35) | 0.005 |
|  |  | Q4 | 1.20 (1.06-1.35) | 0.004 | Q4 | 1.17 (1.03-1.32) | 0.013 | Q4 | 1.17 (1.03-1.32) | 0.013 |
|  |  | Q5 | 1.30 (1.16-1.47) | < 0.001 | Q5 | 1.24 (1.10-1.40) | < 0.001 | Q5 | 1.24 (1.10-1.40) | < 0.001 |
| SIII | H1C0 | Q1 | 1.00 (Reference) | - | Q1 | 1.00 (Reference) | - | Q1 | 1.00 (Reference) | - |
|  |  | Q2 | 0.93 (0.86-1.01) | 0.071 | Q2 | 0.92 (0.85-1.00) | 0.041 | Q2 | 0.92 (0.85-1.00) | 0.041 |
|  |  | Q3 | 0.94 (0.87-1.02) | 0.117 | Q3 | 0.93 (0.86-1.01) | 0.085 | Q3 | 0.93 (0.86-1.01) | 0.085 |
|  |  | Q4 | 0.90 (0.83-0.97) | 0.007 | Q4 | 0.89 (0.82-0.97) | 0.005 | Q4 | 0.89 (0.82-0.97) | 0.005 |
|  |  | Q5 | 0.95 (0.88-1.03) | 0.212 | Q5 | 0.94 (0.87-1.02) | 0.136 | Q5 | 0.94 (0.87-1.02) | 0.138 |
| SIII | H1C1 | Q1 | 1.00 (Reference) | - | Q1 | 1.00 (Reference) | - | Q1 | 1.00 (Reference) | - |
|  |  | Q2 | 1.07 (0.95-1.21) | 0.265 | Q2 | 1.03 (0.91-1.16) | 0.668 | Q2 | 1.03 (0.91-1.16) | 0.667 |
|  |  | Q3 | 1.06 (0.94-1.19) | 0.353 | Q3 | 1.01 (0.89-1.14) | 0.889 | Q3 | 1.01 (0.89-1.14) | 0.894 |
|  |  | Q4 | 1.15 (1.02-1.30) | 0.020 | Q4 | 1.09 (0.96-1.22) | 0.179 | Q4 | 1.08 (0.96-1.22) | 0.188 |
|  |  | Q5 | 1.10 (0.98-1.24) | 0.102 | Q5 | 1.03 (0.91-1.16) | 0.667 | Q5 | 1.02 (0.91-1.16) | 0.690 |
| NLR | H1C0 | Q1 | 1.00 (Reference) | - | Q1 | 1.00 (Reference) | - | Q1 | 1.00 (Reference) | - |
|  |  | Q2 | 0.93 (0.87-1.01) | 0.088 | Q2 | 0.94 (0.87-1.02) | 0.148 | Q2 | 0.94 (0.87-1.02) | 0.149 |
|  |  | Q3 | 0.90 (0.83-0.97) | 0.009 | Q3 | 0.91 (0.85-0.99) | 0.025 | Q3 | 0.91 (0.85-0.99) | 0.025 |
|  |  | Q4 | 0.89 (0.82-0.96) | 0.003 | Q4 | 0.91 (0.84-0.98) | 0.014 | Q4 | 0.91 (0.84-0.98) | 0.014 |
|  |  | Q5 | 0.95 (0.88-1.02) | 0.160 | Q5 | 0.96 (0.89-1.04) | 0.355 | Q5 | 0.96 (0.89-1.04) | 0.354 |
| NLR | H1C1 | Q1 | 1.00 (Reference) | - | Q1 | 1.00 (Reference) | - | Q1 | 1.00 (Reference) | - |
|  |  | Q2 | 0.97 (0.86-1.10) | 0.630 | Q2 | 0.96 (0.85-1.08) | 0.508 | Q2 | 0.96 (0.85-1.08) | 0.505 |
|  |  | Q3 | 0.98 (0.87-1.11) | 0.794 | Q3 | 0.97 (0.86-1.09) | 0.594 | Q3 | 0.97 (0.86-1.09) | 0.587 |
|  |  | Q4 | 1.04 (0.92-1.17) | 0.546 | Q4 | 1.02 (0.90-1.15) | 0.779 | Q4 | 1.02 (0.90-1.14) | 0.796 |
|  |  | Q5 | 1.14 (1.02-1.28) | 0.026 | Q5 | 1.11 (0.99-1.25) | 0.070 | Q5 | 1.11 (0.99-1.25) | 0.076 |
| LMR | H1C0 | Q1 | 1.00 (Reference) | - | Q1 | 1.00 (Reference) | - | Q1 | 1.00 (Reference) | - |
|  |  | Q2 | 0.96 (0.88-1.03) | 0.246 | Q2 | 0.95 (0.88-1.02) | 0.164 | Q2 | 0.95 (0.88-1.02) | 0.163 |
|  |  | Q3 | 0.93 (0.86-1.00) | 0.051 | Q3 | 0.91 (0.84-0.99) | 0.020 | Q3 | 0.91 (0.84-0.99) | 0.019 |
|  |  | Q4 | 0.89 (0.82-0.96) | 0.004 | Q4 | 0.87 (0.81-0.94) | < 0.001 | Q4 | 0.87 (0.81-0.94) | < 0.001 |
|  |  | Q5 | 0.92 (0.85-0.99) | 0.030 | Q5 | 0.88 (0.82-0.95) | 0.002 | Q5 | 0.88 (0.82-0.95) | 0.002 |
| LMR | H1C1 | Q1 | 1.00 (Reference) | - | Q1 | 1.00 (Reference) | - | Q1 | 1.00 (Reference) | - |
|  |  | Q2 | 0.88 (0.78-0.98) | 0.021 | Q2 | 0.87 (0.78-0.98) | 0.019 | Q2 | 0.87 (0.78-0.98) | 0.019 |
|  |  | Q3 | 0.79 (0.70-0.89) | < 0.001 | Q3 | 0.79 (0.70-0.88) | < 0.001 | Q3 | 0.79 (0.70-0.88) | < 0.001 |
|  |  | Q4 | 0.86 (0.77-0.97) | 0.010 | Q4 | 0.86 (0.77-0.97) | 0.011 | Q4 | 0.86 (0.77-0.97) | 0.011 |
|  |  | Q5 | 0.73 (0.65-0.82) | < 0.001 | Q5 | 0.73 (0.64-0.82) | < 0.001 | Q5 | 0.73 (0.65-0.82) | < 0.001 |
| NMR | H1C0 | Q1 | 1.00 (Reference) | - | Q1 | 1.00 (Reference) | - | Q1 | 1.00 (Reference) | - |
|  |  | Q2 | 0.89 (0.83-0.97) | 0.004 | Q2 | 0.89 (0.83-0.96) | 0.004 | Q2 | 0.89 (0.83-0.96) | 0.004 |
|  |  | Q3 | 0.90 (0.83-0.97) | 0.005 | Q3 | 0.89 (0.83-0.97) | 0.004 | Q3 | 0.89 (0.83-0.96) | 0.004 |
|  |  | Q4 | 0.85 (0.78-0.92) | < 0.001 | Q4 | 0.84 (0.78-0.91) | < 0.001 | Q4 | 0.84 (0.78-0.91) | < 0.001 |
|  |  | Q5 | 0.85 (0.79-0.92) | < 0.001 | Q5 | 0.83 (0.77-0.90) | < 0.001 | Q5 | 0.83 (0.77-0.90) | < 0.001 |
| NMR | H1C1 | Q1 | 1.00 (Reference) | - | Q1 | 1.00 (Reference) | - | Q1 | 1.00 (Reference) | - |
|  |  | Q2 | 0.93 (0.82-1.04) | 0.193 | Q2 | 0.92 (0.82-1.03) | 0.150 | Q2 | 0.92 (0.82-1.03) | 0.149 |
|  |  | Q3 | 0.93 (0.83-1.05) | 0.250 | Q3 | 0.93 (0.82-1.04) | 0.193 | Q3 | 0.92 (0.82-1.04) | 0.179 |
|  |  | Q4 | 0.91 (0.81-1.02) | 0.120 | Q4 | 0.89 (0.80-1.01) | 0.062 | Q4 | 0.89 (0.80-1.01) | 0.062 |
|  |  | Q5 | 0.90 (0.80-1.01) | 0.077 | Q5 | 0.87 (0.77-0.98) | 0.018 | Q5 | 0.87 (0.77-0.98) | 0.018 |
| PLR | H1C0 | Q1 | 1.00 (Reference) | - | Q1 | 1.00 (Reference) | - | Q1 | 1.00 (Reference) | - |
|  |  | Q2 | 0.97 (0.90-1.05) | 0.475 | Q2 | 0.95 (0.88-1.03) | 0.250 | Q2 | 0.95 (0.88-1.03) | 0.251 |
|  |  | Q3 | 0.96 (0.89-1.04) | 0.306 | Q3 | 0.95 (0.88-1.03) | 0.179 | Q3 | 0.95 (0.88-1.03) | 0.180 |
|  |  | Q4 | 0.98 (0.90-1.06) | 0.566 | Q4 | 0.97 (0.90-1.05) | 0.501 | Q4 | 0.97 (0.90-1.05) | 0.502 |
|  |  | Q5 | 0.99 (0.92-1.07) | 0.856 | Q5 | 1.02 (0.94-1.10) | 0.643 | Q5 | 1.02 (0.94-1.10) | 0.640 |
| PLR | H1C1 | Q1 | 1.00 (Reference) | - | Q1 | 1.00 (Reference) | - | Q1 | 1.00 (Reference) | - |
|  |  | Q2 | 1.05 (0.93-1.18) | 0.440 | Q2 | 1.00 (0.89-1.13) | 0.996 | Q2 | 1.00 (0.89-1.13) | 0.993 |
|  |  | Q3 | 1.07 (0.95-1.20) | 0.275 | Q3 | 1.02 (0.90-1.14) | 0.797 | Q3 | 1.02 (0.90-1.14) | 0.804 |
|  |  | Q4 | 1.04 (0.93-1.18) | 0.475 | Q4 | 1.00 (0.89-1.13) | 0.991 | Q4 | 1.00 (0.89-1.12) | 0.975 |
|  |  | Q5 | 1.02 (0.91-1.15) | 0.697 | Q5 | 1.01 (0.90-1.14) | 0.872 | Q5 | 1.01 (0.89-1.14) | 0.901 |
| WBC | H1C0 | Q1 | 1.00 (Reference) | - | Q1 | 1.00 (Reference) | - | Q1 | 1.00 (Reference) | - |
|  |  | Q2 | 0.97 (0.89-1.04) | 0.379 | Q2 | 0.95 (0.88-1.03) | 0.207 | Q2 | 0.95 (0.88-1.03) | 0.209 |
|  |  | Q3 | 0.96 (0.89-1.04) | 0.347 | Q3 | 0.94 (0.87-1.02) | 0.114 | Q3 | 0.94 (0.87-1.02) | 0.115 |
|  |  | Q4 | 0.97 (0.90-1.05) | 0.439 | Q4 | 0.94 (0.87-1.01) | 0.103 | Q4 | 0.94 (0.87-1.01) | 0.104 |
|  |  | Q5 | 1.00 (0.92-1.08) | 0.964 | Q5 | 0.95 (0.87-1.02) | 0.172 | Q5 | 0.95 (0.87-1.02) | 0.174 |
| WBC | H1C1 | Q1 | 1.00 (Reference) | - | Q1 | 1.00 (Reference) | - | Q1 | 1.00 (Reference) | - |
|  |  | Q2 | 1.10 (0.97-1.24) | 0.139 | Q2 | 1.07 (0.95-1.20) | 0.295 | Q2 | 1.07 (0.95-1.20) | 0.289 |
|  |  | Q3 | 1.08 (0.95-1.22) | 0.227 | Q3 | 1.03 (0.92-1.17) | 0.596 | Q3 | 1.03 (0.92-1.17) | 0.581 |
|  |  | Q4 | 1.17 (1.04-1.32) | 0.009 | Q4 | 1.10 (0.98-1.24) | 0.103 | Q4 | 1.11 (0.98-1.25) | 0.101 |
|  |  | Q5 | 1.11 (0.98-1.25) | 0.089 | Q5 | 1.01 (0.89-1.14) | 0.901 | Q5 | 1.01 (0.89-1.14) | 0.902 |
| Neutrophil | H1C0 | Q1 | 1.00 (Reference) | - | Q1 | 1.00 (Reference) | - | Q1 | 1.00 (Reference) | - |
|  |  | Q2 | 0.97 (0.90-1.05) | 0.474 | Q2 | 0.98 (0.90-1.05) | 0.524 | Q2 | 0.98 (0.90-1.05) | 0.527 |
|  |  | Q3 | 0.91 (0.84-0.99) | 0.020 | Q3 | 0.91 (0.84-0.98) | 0.017 | Q3 | 0.91 (0.84-0.98) | 0.017 |
|  |  | Q4 | 0.90 (0.83-0.97) | 0.007 | Q4 | 0.89 (0.82-0.96) | 0.003 | Q4 | 0.89 (0.82-0.96) | 0.003 |
|  |  | Q5 | 1.00 (0.92-1.08) | 0.924 | Q5 | 0.97 (0.89-1.05) | 0.403 | Q5 | 0.97 (0.89-1.05) | 0.403 |
| Neutrophil | H1C1 | Q1 | 1.00 (Reference) | - | Q1 | 1.00 (Reference) | - | Q1 | 1.00 (Reference) | - |
|  |  | Q2 | 1.04 (0.92-1.17) | 0.534 | Q2 | 1.03 (0.91-1.16) | 0.611 | Q2 | 1.03 (0.91-1.17) | 0.610 |
|  |  | Q3 | 1.09 (0.97-1.23) | 0.149 | Q3 | 1.07 (0.95-1.20) | 0.293 | Q3 | 1.07 (0.95-1.20) | 0.290 |
|  |  | Q4 | 1.09 (0.97-1.23) | 0.169 | Q4 | 1.04 (0.93-1.18) | 0.480 | Q4 | 1.04 (0.93-1.18) | 0.482 |
|  |  | Q5 | 1.14 (1.01-1.28) | 0.033 | Q5 | 1.05 (0.93-1.19) | 0.400 | Q5 | 1.05 (0.93-1.19) | 0.408 |
| Lymphocyte | H1C0 | Q1 | 1.00 (Reference) | - | Q1 | 1.00 (Reference) | - | Q1 | 1.00 (Reference) | - |
|  |  | Q2 | 0.93 (0.86-1.01) | 0.081 | Q2 | 0.90 (0.83-0.97) | 0.006 | Q2 | 0.90 (0.83-0.97) | 0.006 |
|  |  | Q3 | 0.97 (0.90-1.05) | 0.517 | Q3 | 0.92 (0.85-1.00) | 0.045 | Q3 | 0.92 (0.85-1.00) | 0.045 |
|  |  | Q4 | 0.99 (0.91-1.07) | 0.720 | Q4 | 0.92 (0.85-1.00) | 0.043 | Q4 | 0.92 (0.85-1.00) | 0.043 |
|  |  | Q5 | 0.96 (0.88-1.03) | 0.264 | Q5 | 0.89 (0.82-0.96) | 0.004 | Q5 | 0.89 (0.82-0.96) | 0.004 |
| Lymphocyte | H1C1 | Q1 | 1.00 (Reference) | - | Q1 | 1.00 (Reference) | - | Q1 | 1.00 (Reference) | - |
|  |  | Q2 | 1.03 (0.92-1.16) | 0.566 | Q2 | 0.98 (0.88-1.11) | 0.797 | Q2 | 0.98 (0.87-1.11) | 0.791 |
|  |  | Q3 | 1.04 (0.92-1.17) | 0.548 | Q3 | 0.97 (0.86-1.09) | 0.614 | Q3 | 0.97 (0.86-1.09) | 0.620 |
|  |  | Q4 | 1.02 (0.90-1.14) | 0.805 | Q4 | 0.94 (0.83-1.06) | 0.292 | Q4 | 0.94 (0.83-1.06) | 0.295 |
|  |  | Q5 | 0.96 (0.85-1.08) | 0.499 | Q5 | 0.89 (0.79-1.00) | 0.059 | Q5 | 0.89 (0.79-1.00) | 0.060 |
| Monocyte | H1C0 | Q1 | 1.00 (Reference) | - | Q1 | 1.00 (Reference) | - | Q1 | 1.00 (Reference) | - |
|  |  | Q2 | 1.02 (0.94-1.11) | 0.603 | Q2 | 1.02 (0.94-1.11) | 0.569 | Q2 | 1.02 (0.94-1.11) | 0.569 |
|  |  | Q3 | 1.02 (0.94-1.11) | 0.570 | Q3 | 1.03 (0.95-1.11) | 0.529 | Q3 | 1.03 (0.95-1.11) | 0.529 |
|  |  | Q4 | 1.10 (1.02-1.19) | 0.018 | Q4 | 1.10 (1.01-1.19) | 0.023 | Q4 | 1.10 (1.01-1.19) | 0.023 |
|  |  | Q5 | 1.16 (1.07-1.25) | < 0.001 | Q5 | 1.14 (1.06-1.24) | < 0.001 | Q5 | 1.14 (1.06-1.24) | < 0.001 |
| Monocyte | H1C1 | Q1 | 1.00 (Reference) | - | Q1 | 1.00 (Reference) | - | Q1 | 1.00 (Reference) | - |
|  |  | Q2 | 1.02 (0.90-1.15) | 0.747 | Q2 | 1.01 (0.89-1.14) | 0.870 | Q2 | 1.01 (0.89-1.14) | 0.869 |
|  |  | Q3 | 1.20 (1.06-1.35) | 0.003 | Q3 | 1.18 (1.05-1.33) | 0.007 | Q3 | 1.18 (1.05-1.33) | 0.007 |
|  |  | Q4 | 1.09 (0.97-1.24) | 0.151 | Q4 | 1.06 (0.94-1.20) | 0.316 | Q4 | 1.06 (0.94-1.20) | 0.314 |
|  |  | Q5 | 1.29 (1.14-1.45) | < 0.001 | Q5 | 1.22 (1.08-1.37) | 0.001 | Q5 | 1.22 (1.08-1.37) | 0.001 |
| Eosinophil | H1C0 | Q1 | 1.00 (Reference) | - | Q1 | 1.00 (Reference) | - | Q1 | 1.00 (Reference) | - |
|  |  | Q2 | 0.96 (0.89-1.04) | 0.356 | Q2 | 0.95 (0.88-1.02) | 0.169 | Q2 | 0.95 (0.88-1.02) | 0.167 |
|  |  | Q3 | 0.95 (0.88-1.03) | 0.220 | Q3 | 0.93 (0.86-1.01) | 0.075 | Q3 | 0.93 (0.86-1.01) | 0.074 |
|  |  | Q4 | 0.91 (0.84-0.98) | 0.014 | Q4 | 0.88 (0.81-0.95) | 0.002 | Q4 | 0.88 (0.81-0.95) | 0.002 |
|  |  | Q5 | 0.96 (0.89-1.04) | 0.321 | Q5 | 0.92 (0.85-1.00) | 0.040 | Q5 | 0.92 (0.85-1.00) | 0.040 |
| Eosinophil | H1C1 | Q1 | 1.00 (Reference) | - | Q1 | 1.00 (Reference) | - | Q1 | 1.00 (Reference) | - |
|  |  | Q2 | 0.97 (0.86-1.09) | 0.600 | Q2 | 0.95 (0.84-1.06) | 0.346 | Q2 | 0.95 (0.84-1.06) | 0.345 |
|  |  | Q3 | 0.94 (0.84-1.06) | 0.308 | Q3 | 0.91 (0.81-1.02) | 0.111 | Q3 | 0.91 (0.81-1.02) | 0.118 |
|  |  | Q4 | 0.93 (0.83-1.05) | 0.254 | Q4 | 0.89 (0.79-1.00) | 0.054 | Q4 | 0.89 (0.79-1.00) | 0.057 |
|  |  | Q5 | 0.94 (0.83-1.05) | 0.272 | Q5 | 0.87 (0.77-0.98) | 0.022 | Q5 | 0.87 (0.78-0.98) | 0.024 |
| Basophil | H1C0 | Q1 | 1.00 (Reference) | - | Q1 | 1.00 (Reference) | - | Q1 | 1.00 (Reference) | - |
|  |  | Q2 | 0.94 (0.87-1.02) | 0.137 | Q2 | 0.94 (0.87-1.02) | 0.129 | Q2 | 0.94 (0.87-1.02) | 0.129 |
|  |  | Q3 | 0.91 (0.84-0.99) | 0.022 | Q3 | 0.91 (0.85-0.99) | 0.025 | Q3 | 0.91 (0.85-0.99) | 0.025 |
|  |  | Q4 | 0.94 (0.87-1.01) | 0.089 | Q4 | 0.94 (0.87-1.02) | 0.127 | Q4 | 0.94 (0.87-1.02) | 0.127 |
|  |  | Q5 | 0.90 (0.83-0.97) | 0.009 | Q5 | 0.91 (0.84-0.98) | 0.014 | Q5 | 0.91 (0.84-0.98) | 0.014 |
| Basophil | H1C1 | Q1 | 1.00 (Reference) | - | Q1 | 1.00 (Reference) | - | Q1 | 1.00 (Reference) | - |
|  |  | Q2 | 1.05 (0.94-1.18) | 0.384 | Q2 | 1.05 (0.93-1.17) | 0.437 | Q2 | 1.05 (0.93-1.17) | 0.437 |
|  |  | Q3 | 0.94 (0.84-1.06) | 0.303 | Q3 | 0.94 (0.84-1.06) | 0.299 | Q3 | 0.94 (0.84-1.06) | 0.298 |
|  |  | Q4 | 0.90 (0.80-1.01) | 0.079 | Q4 | 0.90 (0.80-1.02) | 0.098 | Q4 | 0.90 (0.80-1.02) | 0.099 |
|  |  | Q5 | 0.88 (0.78-0.99) | 0.035 | Q5 | 0.89 (0.79-1.00) | 0.046 | Q5 | 0.89 (0.79-1.00) | 0.047 |
| Platelet | H1C0 | Q1 | 1.00 (Reference) | - | Q1 | 1.00 (Reference) | - | Q1 | 1.00 (Reference) | - |
|  |  | Q2 | 0.96 (0.89-1.04) | 0.303 | Q2 | 0.93 (0.86-1.01) | 0.079 | Q2 | 0.93 (0.86-1.01) | 0.079 |
|  |  | Q3 | 1.01 (0.94-1.10) | 0.722 | Q3 | 0.97 (0.90-1.05) | 0.453 | Q3 | 0.97 (0.90-1.05) | 0.453 |
|  |  | Q4 | 0.99 (0.92-1.08) | 0.899 | Q4 | 0.94 (0.87-1.02) | 0.160 | Q4 | 0.94 (0.87-1.02) | 0.160 |
|  |  | Q5 | 0.99 (0.91-1.07) | 0.774 | Q5 | 0.95 (0.87-1.02) | 0.172 | Q5 | 0.95 (0.87-1.02) | 0.173 |
| Platelet | H1C1 | Q1 | 1.00 (Reference) | - | Q1 | 1.00 (Reference) | - | Q1 | 1.00 (Reference) | - |
|  |  | Q2 | 1.03 (0.92-1.17) | 0.578 | Q2 | 0.98 (0.87-1.11) | 0.758 | Q2 | 0.98 (0.87-1.11) | 0.775 |
|  |  | Q3 | 1.07 (0.95-1.21) | 0.234 | Q3 | 0.99 (0.88-1.12) | 0.921 | Q3 | 0.99 (0.88-1.12) | 0.933 |
|  |  | Q4 | 1.10 (0.98-1.24) | 0.099 | Q4 | 1.00 (0.89-1.13) | 0.972 | Q4 | 1.00 (0.89-1.13) | 0.971 |
|  |  | Q5 | 0.99 (0.87-1.11) | 0.825 | Q5 | 0.89 (0.79-1.01) | 0.062 | Q5 | 0.89 (0.79-1.01) | 0.061 |
| hs-CRP | H1C0 | Q1 | 1.00 (Reference) | - | Q1 | 1.00 (Reference) | - | Q1 | 1.00 (Reference) | - |
|  |  | Q2 | 0.93 (0.84-1.02) | 0.105 | Q2 | 0.95 (0.87-1.05) | 0.309 | Q2 | 0.95 (0.87-1.05) | 0.310 |
|  |  | Q3 | 0.86 (0.78-0.94) | 0.002 | Q3 | 0.90 (0.81-0.99) | 0.033 | Q3 | 0.90 (0.81-0.99) | 0.033 |
|  |  | Q4 | 0.81 (0.74-0.88) | < 0.001 | Q4 | 0.87 (0.79-0.96) | 0.004 | Q4 | 0.87 (0.79-0.96) | 0.004 |
|  |  | Q5 | 0.80 (0.73-0.88) | < 0.001 | Q5 | 0.85 (0.77-0.94) | 0.001 | Q5 | 0.85 (0.77-0.94) | 0.001 |
| hs-CRP | H1C1 | Q1 | 1.00 (Reference) | - | Q1 | 1.00 (Reference) | - | Q1 | 1.00 (Reference) | - |
|  |  | Q2 | 0.88 (0.77-1.00) | 0.056 | Q2 | 0.93 (0.81-1.06) | 0.289 | Q2 | 0.93 (0.81-1.06) | 0.283 |
|  |  | Q3 | 0.84 (0.73-0.96) | 0.011 | Q3 | 0.93 (0.80-1.07) | 0.287 | Q3 | 0.93 (0.80-1.07) | 0.285 |
|  |  | Q4 | 0.69 (0.61-0.79) | < 0.001 | Q4 | 0.80 (0.70-0.92) | 0.002 | Q4 | 0.80 (0.70-0.92) | 0.002 |
|  |  | Q5 | 0.63 (0.55-0.73) | < 0.001 | Q5 | 0.71 (0.61-0.82) | < 0.001 | Q5 | 0.71 (0.61-0.82) | < 0.001 |

Supplementary Table 3. Odds Ratios (ORs) for systemic immune-inflammatory markers by HPV and cytology status at baseline among non-current smokers, non-heavy drinkers, and individuals with neither

| **Systemic immune-inflammation markers** | **HPV and cytology group** | **ORs [95% Confidence Interval]** (Q5 *vs*. Q1, Reference: Q1) | | |
| --- | --- | --- | --- | --- |
|  |  | **Non-current smokers** | **Non-heavy drinkers** | **Neither** |
| **SIRI** | H0C0 | Ref | Ref | Ref |
|  | H1C0 | 1.03 [0.94, 1.12] | 0.98 [0.90, 1.07] | 0.98 [0.89, 1.07] |
|  | H1C1 | 1.27 [1.11, 1.46] | 1.31 [1.14, 1.50] | 1.30 [1.12, 1.50] |
| **SIII** | H0C0 | Ref | Ref | Ref |
|  | H1C0 | 0.96 [0.88, 1.05] | 0.93 [0.85, 1.01] | 0.93 [0.85, 1.02] |
|  | H1C1 | 1.07 [0.94, 1.23] | 1.02 [0.89, 1.17] | 1.05 [0.91, 1.21] |
| **NLR** | H0C0 | Ref | Ref | Ref |
|  | H1C0 | 0.99 [0.91, 1.08] | 0.94 [0.87, 1.03] | 0.95 [0.87, 1.04] |
|  | H1C1 | 1.14 [1.00, 1.30] | 1.13 [0.99, 1.29] | 1.13 [0.99, 1.30] |
| **LMR** | H0C0 | Ref | Ref | Ref |
|  | H1C0 | 0.90 [0.82, 0.98] | 0.92 [0.84, 1.00] | 0.93 [0.85, 1.02] |
|  | H1C1 | 0.73 [0.64, 0.84] ^†^ | 0.70 [0.61, 0.80] ^†^ | 0.71 [0.62, 0.82] ^†^ |
| **NMR** | H0C0 | Ref | Ref | Ref |
|  | H1C0 | 0.88 [0.81, 0.96] | 0.85 [0.78, 0.93] ^†^ | 0.86 [0.79, 0.94] ^†^ |
|  | H1C1 | 0.89 [0.78, 1.01] | 0.86 [0.76, 0.98] | 0.87 [0.76, 1.00] |
| **PLR** | H0C0 | Ref | Ref | Ref |
|  | H1C0 | 1.03 [0.95, 1.13] | 1.01 [0.92, 1.10] | 0.99 [0.90, 1.09] |
|  | H1C1 | 1.06 [0.92, 1.21] | 0.96 [0.84, 1.10] | 1.03 [0.89, 1.18] |
| **WBC** | H0C0 | Ref | Ref | Ref |
|  | H1C0 | 0.95 [0.87, 1.04] | 0.94 [0.86, 1.03] | 0.95 [0.87, 1.04] |
|  | H1C1 | 1.03 [0.90, 1.19] | 1.05 [0.92, 1.21] | 1.05 [0.91, 1.21] |
| **Neutrophil** | H0C0 | Ref | Ref | Ref |
|  | H1C0 | 0.99 [0.91, 1.08] | 0.95 [0.87, 1.03] | 0.96 [0.88, 1.05] |
|  | H1C1 | 1.11 [0.97, 1.27] | 1.10 [0.96, 1.26] | 1.12 [0.98, 1.29] |
| **Lymphocyte** | H0C0 | Ref | Ref | Ref |
|  | H1C0 | 0.85 [0.78, 0.93] ^†^ | 0.91 [0.83, 0.99] | 0.89 [0.81, 0.98] |
|  | H1C1 | 0.88 [0.77, 1.01] | 0.90 [0.79, 1.03] | 0.87 [0.75, 1.00] |
| **Monocyte** | H0C0 | Ref | Ref | Ref |
|  | H1C0 | 1.08 [0.99, 1.18] | 1.09 [1.00, 1.19] | 1.06 [0.97, 1.17] |
|  | H1C1 | 1.23 [1.08, 1.41] ^†^ | 1.30 [1.14, 1.49] ^†^ | 1.28 [1.11, 1.47] ^†^ |
| **Eosinophil** | H0C0 | Ref | Ref | Ref |
|  | H1C0 | 0.92 [0.84, 1.00] | 0.91 [0.84, 0.99] | 0.92 [0.84, 1.00] |
|  | H1C1 | 0.90 [0.79, 1.03] | 0.91 [0.79, 1.03] | 0.90 [0.78, 1.03] |
| **Basophil** | H0C0 | Ref | Ref | Ref |
|  | H1C0 | 0.90 [0.83, 0.98] | 0.89 [0.81, 0.97] | 0.88 [0.81, 0.97] |
|  | H1C1 | 0.91 [0.80, 1.04] | 0.92 [0.81, 1.05] | 0.92 [0.81, 1.05] |
| **Platelet** | H0C0 | Ref | Ref | Ref |
|  | H1C0 | 0.94 [0.86, 1.03] | 0.93 [0.85, 1.01] | 0.92 [0.84, 1.01] |
|  | H1C1 | 0.91 [0.79, 1.04] | 0.88 [0.77, 1.00] | 0.89 [0.77, 1.03] |
| **hs-CRP** | H0C0 | Ref | Ref | Ref |
|  | H1C0 | 0.85 [0.76, 0.94] | 0.85 [0.76, 0.95] | 0.86 [0.76, 0.96] |
|  | H1C1 | 0.70 [0.59, 0.83] ^†^ | 0.70 [0.60, 0.83] ^†^ | 0.71 [0.60, 0.85] ^†^ |

Abbreviations: SIRI, systemic inflammation response index; SIII, systemic immune-inflammation index; NLR, neutrophil-to-lymphocyte ratio; LMR, lymphocyte to monocyte ratio; NMR, neutrophil-to-monocyte ratio; PLR, platelet-to-lymphocyte ratio; WBC, white blood cells; hs-CRP, high-sensitivity C-reactive protein; H0C0, HPV-negative and normal cytology; H1C0, HPV-positive and normal cytology; H1C1, HPV-positive and abnormal cytology.

† Indicates statistical significance after applying the Bonferroni correction.
